# Heuristic thinking in the workplace: Evidence from primary care

**DOI:** 10.1002/hec.4534

**Published:** 2022-05-23

**Authors:** Ity Shurtz

**Affiliations:** ^1^ Department of Economics Ben‐Gurion University of the Negev Beer‐Sheva Israel

**Keywords:** attention, heuristic thinking, left digit bias, physician behavior

## Abstract

We study whether primary care physicians (PCPs) exercise left digit bias with respect to patients' age. Relying on a comprehensive administrative visit level data from a large Israeli HMO, we measure the intensity of patients' medical examination in visits that take place around a decadal birthday—a birthday that ends with zero—within a regression discontinuity framework. We find that in standard settings with clear patient information there is no evidence that PCPs exhibit left digit bias. However, when PCPs meet unfamiliar patients seeking immediate care, they are more likely to use basic diagnostic tests just above the decadal birthday threshold, indicating that under these circumstances, PCPs do use left digit bias.

## 1 INTRODUCTION

What role does inattention play in physician decision‐making? Understanding physician decision‐making is key to high‐quality healthcare. This issue has been the focus of a fast‐growing recent line of research.^1^ In settings with decision‐relevant attributes that are not entirely salient, given scarce cognitive resources, individuals tend to pay attention to components of the relevant information as part of their decision‐making process, that is, use heuristics.^2^ It is not well‐understood, however, to what extent physicians use heuristic thinking. In this study, we ask: do primary care physicians (or “PCPs”) use simplifying heuristics in their clinical decision making, and if so, under what circumstances? Concretely, we examine whether PCPs exercise the so‐called left digit bias with respect to the patients' age.

PCPs are a particularly important group of physicians. They play a central role in the delivery of health care in developed countries, accounting for a substantial portion of health care cost—around 14% of total health spending in OECD countries.^3^ As part of their practice, PCPs provide first contact health care, disease diagnosis, maintenance of continuity of care, management of chronic conditions and coordination with other health care providers (Starfield et al., 2005). PCPs typically perform their tasks during short office appointments, spending limited amount of time to each specific topic they address (Tai‐Seale et al., 2007), at a fast work pace (Linzer et al., 2009). It is therefore interesting to examine primary care physicians' heuristic thinking.

Using a simple conceptual framework (Chetty et al., 2009; DellaVigna, 2009; Lacetera et al., 2012), we show that if PCPs exercise left digit bias with respect to the patient's age, namely, if they factor a truncated value of the patient's age into the diagnosis process, the patient's medical examination would be discontinuously more intensive above a decadal birthday—a birthday that ends with zero. To examine this issue, we rely on comprehensive administrative visit level data from a large Israeli HMO (“the HMO”). Using these data, we measure the intensity of patients' medical examination in visits around a decadal birthday by analyzing the utilization of basic diagnostic tests. Diagnostic tests are a commonly used measure of diagnosis intensity and their utilization in the primary care setting is very prevalent—diagnostic tests are needed to establish a diagnosis in over 20% of primary care consultations (Epner et al., 2013; O’Sullivan et al., 2018). In essence, we examine, within a regression discontinuity framework (RDD), if there is evidence that visits that occur just after a decadal birthday are associated with more utilization of basic diagnostic tests. Notably, we are not aware of age‐based guidelines that target populations above decadal birthdays concerning the utilization of the basic diagnostic tests that we analyze here. We explicitly examine this issue below and find no indication that this issue drives our results.

In the HMO, patients are enrolled with a regular primary care physician, with whom they often have a longstanding physician‐patient relationship. Normally, a primary care visit is scheduled with the patient's regular physician. However, if patients request care outside of their physician's regular office hours or when their physician is absent, they are referred to another physician at the clinic, who typically meets them for the first time.^4^ We refer to these two very different, naturally occurring settings as *visits with familiar and unfamiliar patients*. As we explain in more detail below, relative to visits with familiar patients, visits with unfamiliar patients are held with relatively opaque patient information. We, therefore, hypothesize that these visits are more likely to give rise to heuristic thinking.

The RDD analysis shows that physicians are more likely to use diagnostic tools when they meet patients short after a decadal birthday in visits with unfamiliar patients. By contrast, in visits with familiar patients, the effect is small and statistically insignificant. Given statistical power limitations, this difference is not highly significant, yet it shows that the two groups differ in a statistically meaningful sense. These findings support the view that decision‐making context, and specifically background information, play a key role in inducing heuristic thinking among primary care physicians.^5^

We assess whether the increased use of diagnostic tests in visits with unfamiliar patients around decadal birthdays impacted a set of subsequent treatment outcomes which correspond to potential physicians' responses to informative diagnostic tests result. None of the treatment aspects we examine changed around the decadal birthday threshold, suggesting that the increase in the utilization of diagnostic tests around decadal birthdays did not induce overall changes in the course of treatment of affected patients.

Finally, we analyze non‐decadal birthdays—birthdays that do not end with zero. If our former results reflect physicians' left digit bias, we expect to find smaller effects around non‐decadal birthdays. We find no evidence of a significant increase in the utilization of diagnostic tests around all non‐decadal birthdays pooled together nor around any of the non‐decadal birthdays separately. These results indicate that the decadal birthdays' effect is unique, supporting the interpretation of the results as arising from the left digit bias heuristic.

**Interpretation and policy implications.** The evidence suggests two main takeaways. First, we find that in settings with longitudinal physician‐patient relationships and clear patient information, there is no evidence that PCPs exhibit left digit bias. However, when PCPs meet sporadically unfamiliar patients seeking immediate care, they exercise left digit bias with respect to the patients' age. Hence, the analysis of PCP behavior suggests that decision‐making settings matter for the use of heuristic thinking. Particularly, it shows that heuristic thinking tends to emerge in relatively confusing and opaque circumstances when less background information is readily available. This result sheds light on the impact of decision‐making settings on expert choices in medicine and beyond.

Second, a priori, it is not clear if the increase in diagnostic tests is efficient or not. The decadal age threshold may operate as a naturally occurring reminder about the patient's age inducing the physician to order the “age‐appropriate” amount of diagnostic tests. On the other hand, it may prompt unnecessary additional tests. While we cannot determine conclusively whether the additional diagnostic tests indicate “waste”, we find no evidence of impact on subsequent treatment. If one is willing to take our results at face value, then from a health care policy perspective, our findings can be viewed as evidence in favor of developing physician‐patient relationships and care continuity in the primary care setting. The longitudinal physician‐patient relationship appears to reduce the tendency to use heuristic thinking, perhaps because they alleviate some physician cognitive load.

**Literature.** A growing body of literature studies the consequences of heuristics and limited attention in healthcare. While health care consumers' inattention has been studied quite extensively, much less is known about the role of limited attention and heuristic thinking in physician decision making. Existing work on this issue include Rizzo and Zeckhauser (2003) that find that physicians respond to loss aversion with respect to reference income by engaging in income generating activities, including changing practice style. Frank and Zeckhauser (2007) show that drug prescribing patterns are consistent with the use of ready‐to‐wear treatments, namely, physicians have a small number of favorite drugs that they prescribe to most patients with a given condition.^6^

Two recent studies examine similar issues in the hospital setting.^7^ Olenski et al. (2020) study physicians' left digit bias in the context of Coronary‐Artery Bypass Graft Surgery (CABG). They show that patients admitted within 2 weeks after their 80th birthday were significantly less likely to undergo CABG than those admitted 2 weeks or less before their 80th birthday. Coussens (2018) examines the use of heuristics in the emergency department. He finds evidence that patients arriving in the ED just after their 40th birthday are 20% more likely to be diagnosed with ischemic heart disease relative to patients arriving just before it, leading to a reduction in the number of missed IHD diagnoses. These studies show that in the context of the ED and CABG, heuristic thinking may have important consequences for patient health. These results differ from our findings for the primary care setting, but these differences are not surprising and the overall picture reinforces the takeaway of this paper. In more “difficult” settings such as the hospital environment, or, in our case, visits with unfamiliar patients, where a prior physician‐patient relationship does not typically exist, and background information is scarce, heuristic thinking is more likely to arise.

The remainder of the paper is structured as follows. Section 2 lays out a simple framework and describes the empirical strategy, Section 3 describes the data and presents the empirical analysis, and Section 4 concludes.

## 2 FRAMEWORK AND EMPIRICAL STRATEGY

The physician interface at the HMO shows the patient's age at the date of the visit on the header of the patient's electronic medical record. Age is represented in a somewhat unique format in terms of years and months separated by a dot.^8^ For example, the age of a patient that was born on August 15, 1962, and visits the physician on August 1, 2012, would be 49.11, that is, 49 and 11 months^9^ If that same patient were to visit the physician 1 month later, on September 1, 2012, her age, as it appears on the header, would be 50.00.

We use a simple framework to describe physician inattention to the patient's exact age [see Lacetera et al. (2012) and Chetty et al. (2009)]. The underlying assumption here is that the physician focuses on the leftmost digit of the patient's age and is inattentive to the digits farther to the right. Let *age* ∈ [29.00, 89.11] be a patient's age—as it appears in the patient's electronic medical record. Assume that physician perceives the patient's age as:

(1) $$\hat{age}=d_{1}\cdot 10+\left(1-\theta \right)\cdot \left(d_{2}+\frac{d_{3}\cdot 10+d_{4}}{12}\right)$$

where *d*_*i*_ is the value of the digit at location *i* of *age* and *θ* ∈ [0, 1] is the inattention parameter. Hence, physicians take into account the truncated value of the patient's age in full and are less attentive to the exact patient's age. The physician perceives a 1‐year change in *age* that does not involve a change in the left digit as a change of 1 − *θ* in the patient's age. A 1‐year change in *age* that involves a change in the left digit aligns the perceived age, $\hat{age}$, and the actual age creating a discontinuous jump in the perceived age. Suppose that *θ* = 1. The physician perceives a 69.11 years old patient as a patient in his “sixties”, and the perception of a 70.00 year‐old patient, who is roughly the same age, jumps to be that of a patient in his “seventies”.

Consider a physician that chooses an action *a* given a patient's perceived age denoted by $\hat{age}$

(2) $$\underset{a}{\max\;}u\left(a,\hat{age}\right)$$

Assuming that other things being equal, older age is associated with more conservative medical examination, this simple framework predicts that a 1‐year age increase when only one digit changes (e.g., 68.11–69.00) would induce a smaller effect on physician behavior than that of a 1‐year age increase around a decadal birthday (e.g., 69.11–70.00). A change in the left digit of a patient's age would induce a discontinuously more conservative physician examination behavior. Specifically, we hypothesize that there would be a discontinuous increase in the amount of basic diagnostic tests that physicians use as part of the medical examination of a patient just above a decadal age relative to a patient just below it.

In order to empirically examine this hypothesis we implement an RDD approach. Let *τ* be the number of days relative to the closest birthday (in absolute terms) at the time of the visit to the clinic. Let the treatment indicator *D* equal 1 if the visit took place less than 6 months after the patient's birthday (in which case *τ* ≥ 0), and 0 otherwise. Consider the following model (Angrist and Pischke (2008)):

(3) $$y=\alpha_{0}+\beta_{0}D+f\left(\tau \right)+\epsilon$$

where *y* is an outcome variable that measures the intensity of the physical examination, such as an indicator for using basic diagnostic tests during a visit at the clinic. *f*(*τ*) is a completely flexible control function, and is continuous at *τ* = 0. The parameter of interest in this model is the coefficient *β*_0_, which measures the causal effect of visiting the clinic just after the patient's birthday rather than right before it on the intensity of the medical examination. Intuitively, given that *f*(*τ*) absorbs any continuous relationship between the timing of the visit relative to the patient's birthday and the outcome variable, the coefficient *β*_0_ estimates the discontinuous relations between visiting the clinic after the patient's birthday and the outcome variable. Therefore, we may attribute its estimates to the causal effect of a (decadal) birthday on the intensity of the medical examination. We estimate the model applying standard regression discontinuity design methods as we describe in detail below.

## 3 EMPIRICAL ANALYSIS

Our analysis draws on data from a detailed administrative visit‐level database covering all primary care visits in 11 clinics in the Jerusalem area of the HMO—one of four HMOs that provide the vast majority of primary care in the country—in 2011–2014.^10^ The HMO's patients are enrolled with a regular primary care physician at their local clinic. Patients may choose their regular PCP every quarter, but in practice, there is little movement across physicians. By default, the regular PCP is the main point of contact with the healthcare system. However, patients may meet other physicians at the clinic if they drop in or contact the clinic with urgent medical issues when their regular physician is unavailable.

We distinguish between two very different, naturally occurring settings. The first is visits with familiar patients—patients who meet the physician they are enrolled with—the standard and more common visit type. About 74% of the visits belong to this visit type. Patients meet their regular physician quite frequently, and they often have a longstanding physician‐patient relationship. In the clinics we study, the median number of times a patient meets her physician in a year is three. These visits are arguably held in a clear and well‐understood context. Due to the longitudinal physician‐patient relationship, background information about the patient is readily available to the physician. Furthermore, the physician‐patient interaction is characterized by trust and a sense of responsibility (Saultz, 2003). The second is visits with unfamiliar patients, where patients who seek immediate care meet, usually at their local clinic, physicians with whom they typically had no previous contact. The absence of prior physician‐patient contact and the sporadic nature of the visit limit the availability of patient information and naturally eliminate the preexisting sense of physician‐patient bonding. Relative to visits with familiar patients, the context of this visit type is less clear and standard with relatively opaque patient information. We examine whether heuristic thinking arises in these two very different, naturally occurring settings. We hypothesize that the latter visit type is more likely to give rise to heuristic thinking.

The two visit types differ in other ways because patients self‐select to visits with physicians they are not enrolled with. By definition, such visits occur when patients require care and their physician is unavailable. Therefore, the two visit types may differ from one another in terms of patient characteristics and condition, as shown below. Nonetheless, our identification strategy relies on the assumption that while patients self‐select to visits with physicians they are not enrolled with, the timing of their visit relative to their exact decadal birthday is random. Namely, patients do not systematically time their visit to an unfamiliar physician to the weeks before and after a decadal birthday. This assumption cannot be fully tested. However, in what follows, we perform a thorough examination of its validity.

### 3.1 Data

The visit‐level data include physician and patient identifiers, patient characteristics such as gender, age, country of origin, chronic conditions, and a full summary of the visit, including referrals to laboratory tests, imaging, and prescriptions. Table 1 provides descriptive statistics regarding these face‐to‐face visits by visit type. In the final sample, we include visits within 180 days relative to the closest decadal birthday. To exclude follow‐up visits, we keep only visits that occurred more than 30 days since the previous visit to the clinic. There are 7098 (25,695) such office visits with unfamiliar (familiar) patients made by 5532 (12,290) patients to 96 (77) physicians.^11^ In 78% of visits with familiar patients, patients meet the physician at least once in the 360 days before the visit, relative to 22% in visits with unfamiliar patients. Namely, as expected, in the familiar patients' visits, patients are much more likely to have prior contact with the physician.^12^ Additionally, patients in the unfamiliar group are less likely to meet any physician in the 90 days before the visit.

The table further indicates that visits with unfamiliar patients involve younger and healthier patients. These visits tend to have a higher share of acute conditions like upper respiratory or viral infections. There is also less utilization of diagnostic tests in these visits. This is consistent with a selection process whereby younger and healthier patients with acute medical issues are less flexible about the timing of the office visit and care less about the identity of the available physician, while sicker patients are more flexible toward office visit timing and care more about meeting their “own” physician.

**TABLE 1:** Summary statistics

	Unfamiliar		Familiar	
	Mean	SD	Mean	SD
	(1)	(2)	(3)	(4)
Mean age	46.30	15.75	52.68	16.64
Share women	0.59	0.49	0.58	0.49
Share born in Israel	0.69	0.46	0.69	0.46
Share hypertension	0.21	0.41	0.32	0.47
Share smokers	0.37	0.48	0.38	0.49
Share hyperlipidemia	0.35	0.48	0.47	0.50
Share asthma	0.06	0.24	0.07	0.25
Share overweight	0.22	0.41	0.28	0.45
Share diabetes	0.12	0.32	0.18	0.38
Share referral to X‐ray	0.04	0.19	0.05	0.21
Share referral to blood test	0.07	0.26	0.16	0.37
Share referral to urine test	0.02	0.13	0.04	0.19
Share referral to basic diagnostic test	0.12	0.33	0.23	0.42
Share upper respiratory infection acute	0.04	0.20	0.02	0.15
Share upper respiratory tract infection	0.03	0.18	0.01	0.12
Share viral infection unspecified	0.02	0.15	0.01	0.12
Share visit in prior 360 days	0.22	0.42	0.78	0.42
Share visit any physician in prior 90 days	0.33	0.47	0.47	0.50
Number of patients	5532		12,290	
Number of physicians	96		77	
Observations	7098		25,695	

*Note*: The table includes office visits in the clinics used in this study in the period 2011–2014 by visit type (see text).

**FIGURE 1:**
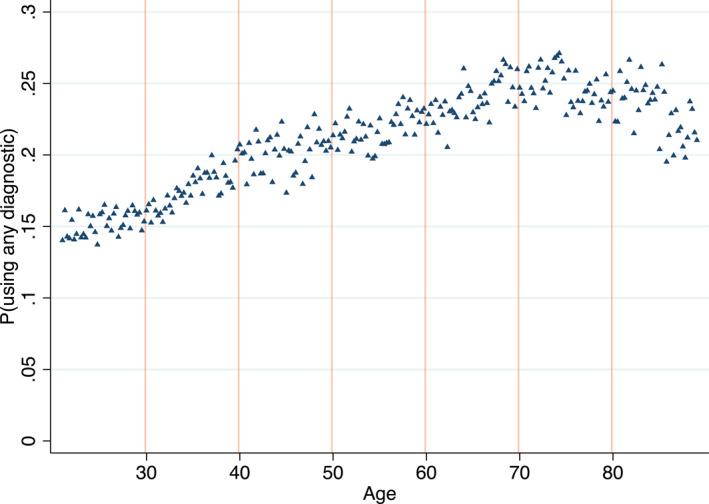
Share of diagnostic tests by age, all visit types. The figure plots the share of visits with any basic diagnostic tests, by age in quarters

The main outcome variable we analyze in order to measure the intensity of patients' medical examination is an indicator for using any of the three most common visit‐level basic diagnostic tests: blood test, X‐ray, and urine test. These tests are relatively inexpensive, often available at the clinic, and give same‐day results. This is not true, for example, in ultrasound scans that often involve several weeks of waiting. Other advanced imaging tools such as CT and MRI scans are rarely used in our setting, and including them does not affect the results. As Table 1 shows, the likelihood of using any of the basic diagnostic tests in visits with unfamiliar (familiar) patients is 12 (23) percent. Figure 1 displays the likelihood of using basic diagnostic tests during office visits by patient age.^13^ Interestingly, the use of diagnostic tests is not monotonic. It increases with age until around age 65, and it starts to decline afterward, forming an “inverse u” shape. The raw data does not exhibit apparent jumps in the use of diagnostics around each of the decadal birthdays separately. Our empirical approach, however, pools together the information from the multiple decadal birthday thresholds to estimate the decadal birthdays' combined effect more precisely (Cattaneo et al., 2016).

**FIGURE 2:**
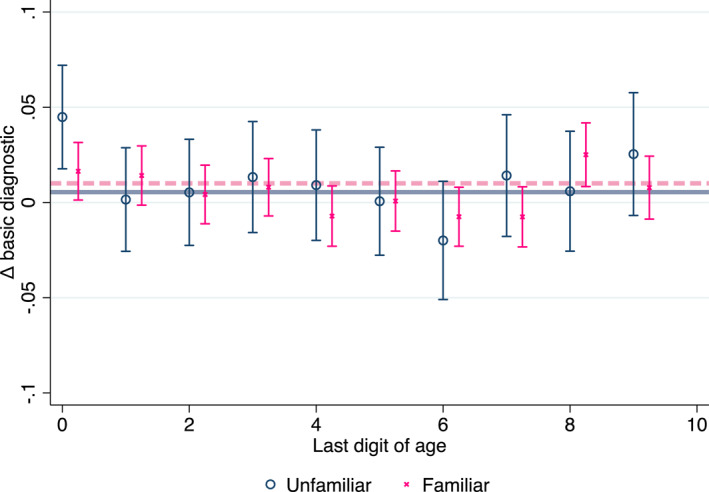
Δ utilization of basic diagnostic tests past birthdays. The figure plots the difference in the likelihood of using any basic diagnostic test between the periods of 29 days after a patient's birthday and 29 days before it. The horizontal solid gray line and the dashed pink line are the average of the respective points for unfamiliar and familiar patients. Vertical spiked lines represent the 95% confidence intervals

Before turning to the RDD analysis, Figure 2 provides some descriptive analysis of the data. The figure displays the difference in the average of our main outcome variable, basic diagnostic tests, between visits that occur 29 days after a patient's birthday and 29 days before it. The hollow gray circles and pink x's display this difference for visits with unfamiliar and familiar patients, respectively. We include only physicians that see regular patients at least 25% of the time to avoid differences in physician composition between the 2 groups. The solid (gray) and dashed (pink) lines represent the averages of the unfamiliar and familiar respective points. Focusing on the gray circles—visits with unfamiliar patients around decadal birthdays, there is a significant 4.5 percentage points increase in the likelihood to use basic diagnostic tests. None of the differences around other birthdays show a similar increase and they are all statistically insignificant. Turning to visits with familiar patients, the pink x's, the change in the use of basic diagnostics around all birthdays, including decadal birthdays is quite small and statistically insignificant (except around birthdays ending with 8). Overall, this figure demonstrates the main result of this paper. In visits with unfamiliar patients, the use of diagnostic tests after decadal birthdays increases and this increase is unique—it is not apparent around other birthdays and in visits with familiar patients.

### 3.2 Visits with familiar and unfamiliar patients

In this section, we report our findings regarding the effect of patients' decadal birthdays on utilization of basic diagnostic tests for the two visit types we describe above—visits with familiar and unfamiliar patients.^14^

**FIGURE 3:**
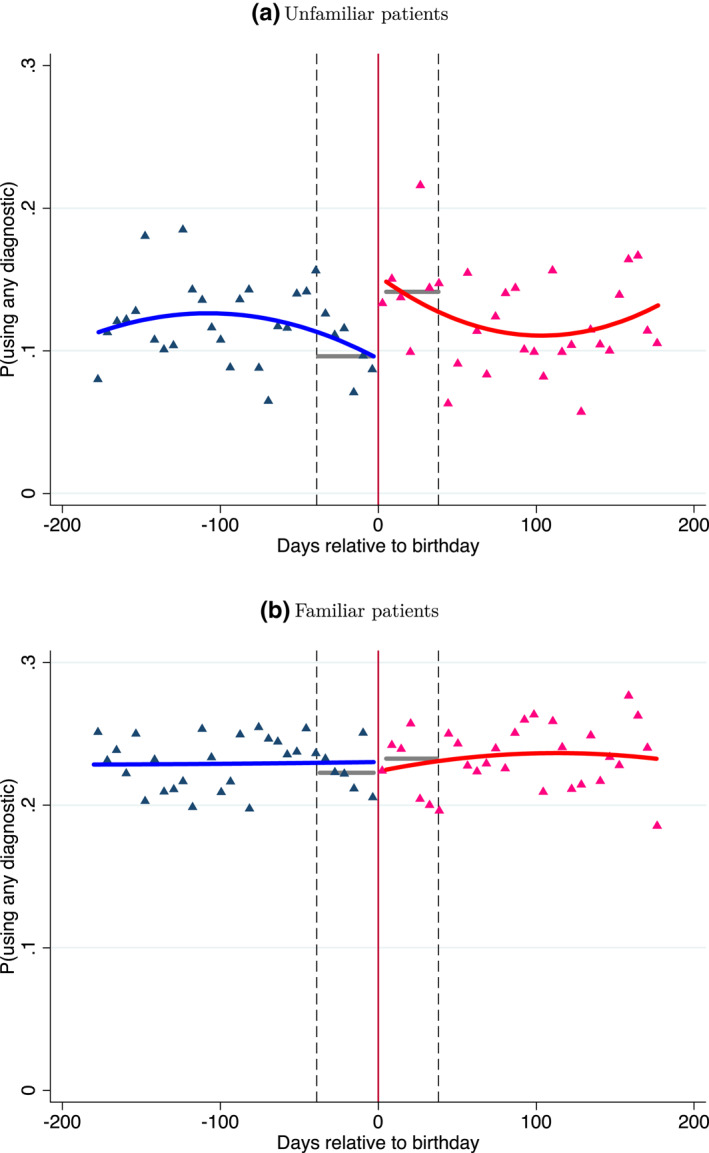
Utilization of basic diagnostic tests around decadal birthdays. Panels (a) and (b) of the figure plot the likelihood of using any basic diagnostic test, by days elapsed relative to a patient's nearest decadal birthday, 180 days before and 180 days after the decadal birthday, in 6 days bins, for visits with unfamiliar and familiar patients, respectively. The vertical solid line represents the decadal birthday threshold. A zero‐order polynomial (gray solid line) is fit within the optimal bandwidth (vertical dashed lines)

Panels (a) and (b) of Figure 3 illustrate the effect visually. They plot the likelihood of using basic diagnostic tests during office visits of unfamiliar and familiar patients, respectively, against the running variable—days elapsed relative to a patient's nearest decadal birthday, 180 days before and after the decadal birthday, in 6‐day bins. We fit two quadratic regression models to the data separately, one below the decadal birthday threshold and one above it.^15^ As panel (a) of the figure illustrates, the decadal birthday threshold shows a 4.5 pp increase in the likelihood to use basic diagnostics in unfamiliar patients' visits. By contrast, there appears to be a very small effect of about 1 pp in the familiar patients' visits even though the outcome's baseline level is almost twice as large in that group.

To quantify this effect numerically, we estimate the model in Equation (3) using a local linear regression analysis with a triangular kernel and mean‐squared error (MSE) optimal bandwidth [see Imbens and Kalyanaraman (2012)]. Since there is no reason to expect a slope in the outcome variable around the cutoff, we use a zero‐order polynomial to avoid overfitting the data.^16^ Columns (1) and (2) of Table 2 report the corresponding estimates [*β*_0_ in Equation (3)] for visits with unfamiliar and familiar patients, respectively. We add a graphical representation of the point estimation to Figure 3 where a polynomial of order zero is fit within the optimal bandwidth (marked by the vertical dashed lines). Consistent with the visual impression, the table shows a significant 4.5 pp estimate in visits with unfamiliar patient. Visits with familiar patients show an insignificant 1 pp increase.

**TABLE 2:** The effect of decadal birthdays on utilization of basic diagnostic tests

	Unfamiliar	Familiar
	(1)	(2)
RDD estimate	0.045**	0.009
	(0.016)	(0.011)
Bandwidth	39	39
Effective observations	1559	5516
Observations	7098	25,695

*Note*: This table provides the RDD estimates of the likelihood to use basic diagnostic tests as per Equation (3). One or two asterisks indicate significance at 5% or 1%, respectively.

**FIGURE 4:**
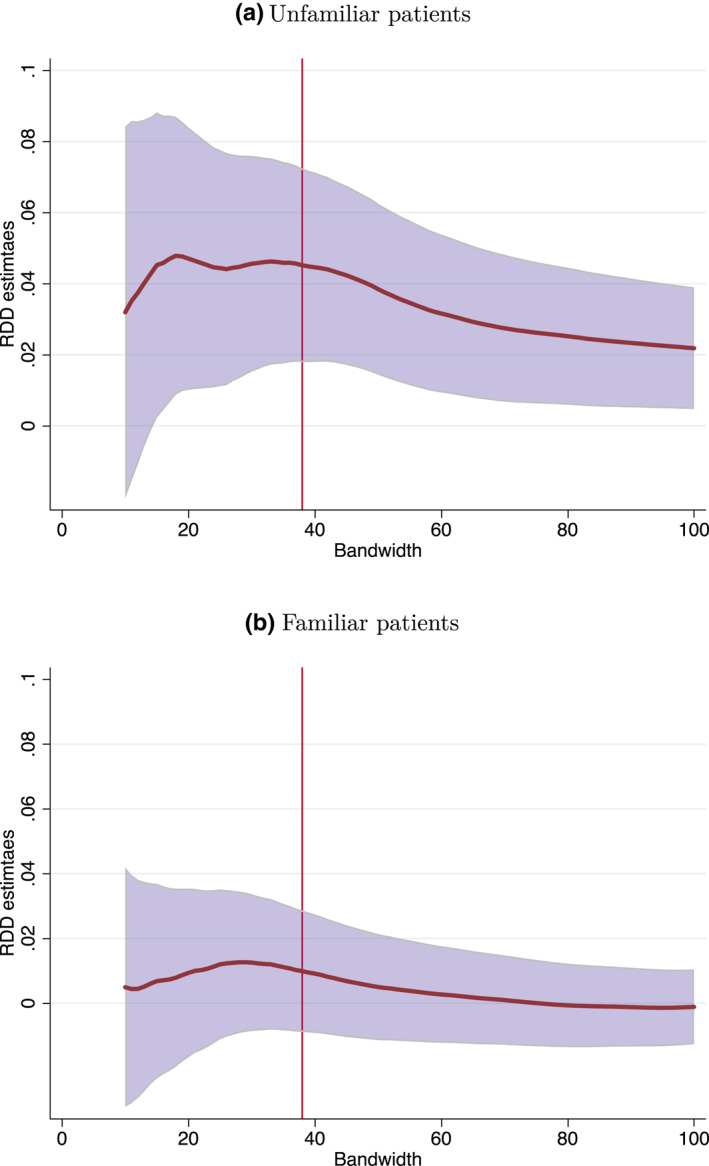
Utilization of basic diagnostic tests around decadal birthdays, varying bandwidths. Panel (a) and (b) plot the RDD estimates of Equation (3) by bandwidth, for visits with unfamiliar and familiar patients, respectively. The light blue area represents the 90% confidence intervals around the estimates

To examine the sensitivity of these results to the choice of bandwidth, we run the regressions using any bandwidth between 10 and 100 days around a decadal birthday. Figure 4 reports the results. As panel (a) of the figure shows, around the optimal bandwidth of 39 days, the unfamiliar patients results appear to be quite stable with statistically significant estimates of about 4 pp. As we increase the bandwidth toward a hundred days, the estimates decrease to about 2 pp, yet they remain statistically significant. The familiar patients results are small and statistically insignificant for any bandwidth.^17^ Given that basic diagnostics are used in about 12% of visits with unfamiliar patients, the results for visits with unfamiliar patients reflect an increase in the likelihood of using basic diagnostic tests in the range 37%‐16%, depending on the bandwidth used. Thus, while the results are qualitatively similar and statistically significant for any bandwidth, the exact magnitude of the point estimates is sensitive to the choice of bandwidth.^18^

### 3.3 Selection checks

Our identification assumption is based on the premise that the timing of office visits around decadal birthdays is as good as random. One threat to identification is that patients may “manipulate the threshold”, namely, time their arrival at the clinic systematically around their decadal birthday. Such selection may arise if, for example, *patients* exercise left digit heuristics. If patients' perception of their age is described by Equation (1), then upon reaching a decadal birthday, they may tend to go to see a physician more (or less) frequently. We examine this issue by testing whether the number of observations (office visits) below the threshold is different from the number of observations above it. If the number of visits changes abruptly around the decadal birthday threshold, this would indicate that patients “manipulate the threshold,” and our identification assumption would lose credibility. To formally test this issue, we follow Cattaneo et al. (2019) and McCrary (2008).

**FIGURE 5:**
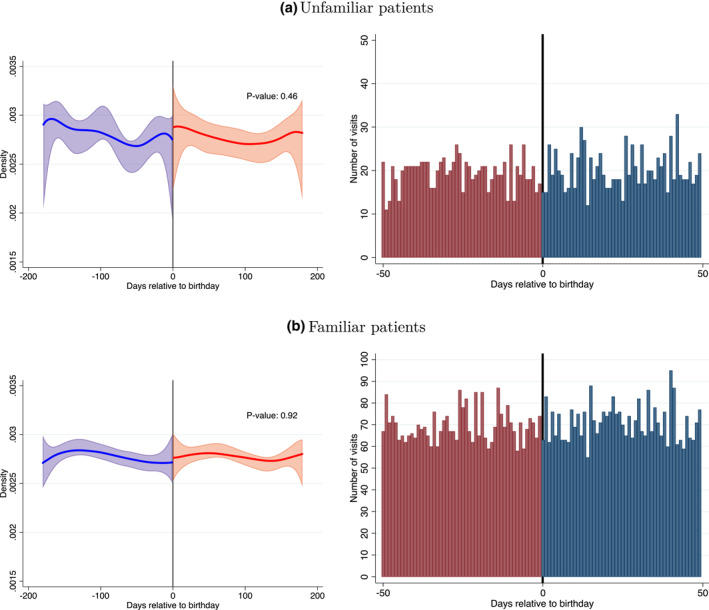
Visit density around decadal birthdays. Panels (a) and (b) of the figure plot the density tests for visits with unfamiliar and familiar patients, respectively. The figure on the right shows number of visits by days relative to a patient's nearest decadal birthday, 50 days before and 50 days after the decadal birthday. The vertical solid line represents the decadal birthday threshold. The figure on the left plots the estimated visit density 180 days before and 180 days after the decadal birthday

Panels (a) and (b) of Figure 5 provide a graphical representation of the test of the continuity in the density around the decadal birthday threshold for visits with unfamiliar and familiar patients respectively. They display the “raw” histogram of the data in a radius of 50 days around the threshold and the visit density estimates using local polynomial density estimations and their 95% confidence intervals. As the figure shows, in both panels, the raw data appear to trend smoothly around the threshold, and the density estimates from both sides of the threshold are very close to each other, and the confidence intervals overlap. Consistent with this impression, the formal tests of the null hypothesis that the density of the running variable is continuous at the decadal birthday threshold can not be rejected for the two visit types.

We further explore this issue by examining systematic differences in observable predetermined characteristics around the decadal age threshold in the two visit types. If patients self‐select to either side of the decadal age threshold, this may be manifested in a discontinuity in one or more of the observable patient characteristics. We, therefore, examine the six most common chronic patient conditions coded in the data to test for discontinuities in their prevalence around the decadal birthday threshold. Additionally, to test for patient selection which is not captured by clinical characteristics, we add a seventh variable: the number of visits to the clinic in the prior 90 days. This variable may identify selection in the dimension of healthcare consumption habits. We report the results of this analysis, derived using the same methodology as the main results, in Table 3. The corresponding figures are shown in the appendix (Appendix Figures A5 and A6 in the Supporting Information S1). The table and figures show no indication of a sharp change in observable patient characteristics in either visit type.

**TABLE 3:** Selection on observables

	Hypertension	Smoking	Asthma	Hyperlipidemia	Obesity	Diabetes	Visit history
	(1)	(2)	(3)	(4)	(5)	(6)	(7)
(a) Unfamiliar							
RDD estimate	0.014	0.004	0.009	−0.017	−0.004	−0.005	0.043
	(0.023)	(0.029)	(0.014)	(0.026)	(0.028)	(0.026)	(0.053)
Bandwidth	32	29	29	33	24	17	30
Effective observations	1277	1149	1149	1314	938	664	1189
Observations	7098	7098	7098	7098	7098	7098	7098
(b) Familiar							
RDD estimate	−0.003	−0.018	0.009	−0.007	−0.012	0.016	0.032
	(0.014)	(0.015)	(0.008)	(0.015)	(0.015)	(0.016)	(0.034)
Bandwidth	32	29	29	33	24	17	30
Effective observations	4540	4106	4106	4665	3396	2365	4252
Observations	25,695	25,695	25,695	25,695	25,695	25,695	25,695

*Note*: This table provides the RDD estimates for the analysis of selection on observables. One or two asterisks indicate significance at 5% or 1%, respectively.

### 3.4 Difference‐in‐discontinuities analysis

We want to assess if the effect we find in visits with unfamiliar patients is larger than that of visits with familiar patients in a statistically meaningful sense. To this end, we use the following difference‐in‐discontinuities model:

(4) $$y=\alpha_{0}+\alpha_{1}\cdot unfamiliar+\beta_{0}\cdot D+\beta_{1}\cdot D\cdot unfamiliar+\epsilon .$$

The coefficient *β*_1_ captures the difference in the effect of decadal birthdays across the two visit types. We report these estimates in Table 4. The 3.5 percentage points difference (s.e. 0.023) is not highly significant, but it allows rejecting the one‐sided null that the difference between the groups is zero with a *p*‐value of 6.2%.^19^ Despite the power limitation, this result shows that the difference between the two groups is statistically valid.

We interpret our result as showing that when physicians encounter unfamiliar patients, they use left digit bias. However, an alternative interpretation would be that the difference arises from cross‐sectional differences in physician response to the threshold. Namely, some physicians tend more to meet unfamiliar patients, and these physicians are also more likely to exhibit heuristic thinking. We want to examine this issue. If cross‐sectional differences between physicians drive the difference between the two visit types, then introducing physician fixed effects to the DD‐RD analysis should absorb this result. Column (2) reports the results of the DD‐RD analysis with physician (and year and month) fixed effects, holding the bandwidth fixed. The estimate is 3 pp, and the *p* value of the one‐sided null increases to 10%. While the results are smaller and less significant, they remain similar, suggesting that they are not driven by differences across physicians but by the type of patients they meet. As expected, controlling for patient characteristics has a very small effect on the results (column (3)).^20^

**TABLE 4:** The effect of decadal birthdays on utilization of basic diagnostic tests, difference‐in‐discontinuities

	(1)	(2)	(3)
DD‐RD estimate	0.035	0.030	0.029
	(0.023)	(0.023)	(0.023)
Physician FEs	No	Yes	Yes
Time FEs	No	No	Yes
Patient characteristics	No	No	Yes
Bandwidth	38	38	38
Effective observations	6884	6884	6884
Observations	32,793	32,793	32,793

*Note*: This table provides the DD—RDD estimates of the likelihood to use basic diagnostic tests as per Equation (3). Time fixed effects include year and month‐of‐year fixed effects. The patient characteristics which are included are: cubic polynomial of age, gender, and the following chronic conditions: hypertension, smoking, asthma, hyperlipidemia, obesity and diabetes. One or two asterisks indicate significance at 5% or 1%, respectively.

An additional concern that arises in our setting is that patients may behave differently exactly on their birthday, for example, they prefer not to visit the clinic on their decadal birthday because they have special plans for that day that change their normal schedule. This issue may imply that patients who arrive at the clinic exactly on their birthday are different from those who do not, which may influence the results. To examine this concern, we perform a “donut hole” analysis.^21^ Namely, we examine the sensitivity of the results to the elimination of observations very close to the decadal birthday threshold. If our results are driven by visits that occur exactly on the birthday or very close to it, we might worry that they reflect a mere “birthday effect”. Table 5 reports the estimation results that omit observations in radii 2 and 4 days around a decadal birthday, respectively. As the results indicate, the estimates appear to be slightly more pronounced than the baseline estimates in Table 2. The effect of omitting 2 and 4 days around a decadal birthday in the unfamiliar patients' visits is 5.3 and 5.6 pp, respectively. The effect remains small and insignificant in the familiar patients' visits, and the DD‐RD estimates are 4.1 and 4.8 pp.

**TABLE 5:** The effect of decadal birthdays, donut hole estimates

	Unfamiliar		Familiar		Diff	
Radius of omitted days	Two	Four	Two	Four	Two	Four
Around birthday	(1)	(2)	(3)	(4)	(5)	(6)
RDD estimate	0.053**	0.056**	0.012	0.008	0.041	0.048*
	(0.017)	(0.017)	(0.012)	(0.012)	(0.023)	(0.024)
Bandwidth	39	39	39	39	39	39
Effective observations	1497	1413	5232	4950	6729	6363
Observations	7098	7098	25,695	25,695	32,793	32,793

*Note*: This table shows RDD estimates for the analysis of the “donut hole” analysis. One or two asterisks indicate significance at 5% or 1%, respectively.

### 3.5 Age‐based guidelines

Another issue to keep in mind is that PCPs sometimes work with age‐based guidelines, mostly for preventive care purposes. Such guidelines introduce simplifying rules of thumb that may induce “coarseness” to physicians' decision‐making. For example, it is recommended for patients above age 50 to perform an occult blood test annually. Therefore, one may expect a sharp increase in the utilization of that test just above age 50, even though colon cancer risk rises smoothly with age with no discontinuous “jump” in risk upon turning 50.

The use of age‐based guidelines should be more relevant for physicians that meet with their regular patients and provide preventive care. It is less likely to arise in the context of unfamiliar patients, where patients seek immediate care by a physician they are not enrolled with. Nevertheless, we now take a closer look at this issue for both visit types and assess if our results have to do with such guidelines.

While we are not aware of guidelines involving our basic diagnostic tools that explicitly coincide with decadal birthdays, one recommendation that might induce such interaction for blood tests is to measure cholesterol periodically. It is recommended to measure blood cholesterol levels every five years, starting at age 35 for men and 40 for women. Hence, it could be the case that physicians tend to prescribe this test when they meet patients just after decadal birthdays.

We cannot observe blood cholesterol tests separately. However, the data allow us to distinguish between two types of blood tests. The first, blood biochemistry, measures certain chemicals in the blood. It includes the cholesterol test and also blood sugar level, electrolytes, creatinine, and uric acid. These tests provide information about general health and on the function of organs like the liver and kidneys. The second, blood hematology, includes blood count, a common blood test to detect infections and other disorders, which is often prescribed in acute conditions. To take a closer look at this issue, we run the analysis again, excluding blood biochemistry. These two types of blood tests are often taken together. Hence, this breakdown of the outcome variable is a bit coarse. However, if cholesterol tests, as opposed to diagnoses of acute conditions, are driving our baseline results, this exercise should generate weaker results. Panel (a) of Table 6 shows the results of this regression. The estimates are very similar to the results reported in Table 2.

**TABLE 6:** The effect of decadal birthdays by diagnostic tool

	Unfamiliar	Familiar	DD‐RD	
	(1)	(2)	(3)	(4)
(a) All no bio‐chem				
RDD estimate	0.048**	0.009	0.039	0.029
	(0.018)	(0.012)	(0.025)	(0.026)
Bandwidth	29	29	29	29
Effective observations	1149	4106	5255	5255
(b) Blood test				
RDD estimate	0.035*	0.003	0.032	0.026
	(0.017)	(0.013)	(0.027)	(0.028)
Bandwidth	24	24	24	24
Effective observations	938	3396	4334	4334
(c) X‐ray				
RDD estimate	0.017	0.004	0.013	0.013
	(0.009)	(0.005)	(0.011)	(0.011)
Bandwidth	44	44	44	44
Effective observations	1776	6204	7980	7980
(d) Urine test				
RDD estimate	0.006	−0.003	0.009	0.010
	(0.007)	(0.005)	(0.009)	(0.009)
Bandwidth	47	47	47	47
Effective observations	1886	6609	8495	8495
(e) Blood bio‐chem				
RDD estimate	0.025	−0.001	0.026	0.021
	(0.015)	(0.013)	(0.025)	(0.026)
Bandwidth	25	25	25	25
Effective observations	971	3550	4521	4521
(f) Blood no bio‐chem				
RDD estimate	0.037*	−0.000	0.037	0.029
	(0.017)	(0.013)	(0.027)	(0.027)
Bandwidth	21	21	21	21
Effective observations	825	2951	3776	3776
Controls	No	Yes	No	Yes
Observations	7098	25,695	32,793	32,793

*Note*: This table provides the RDD estimates of the number of diagnoses as per Equation (3). Controls include physician fixed effects; time fixed effects, which include year and month‐of‐year fixed effects; patient characteristics: cubic polynomial of age, gender, and the following chronic conditions: hypertension, smoking, asthma, hyperlipidemia, obesity and diabetes. One or two asterisks indicate significance at 5% or 1%, respectively.

Next, we analyze the outcome variable element by element. Panels (b)‐(d) report the results for blood tests, X‐rays, and urine tests, respectively. While the estimates are smaller and lose their statistical significance, the unfamiliar patients' results are all positive and larger than those of familiar patients, consistent with the gist of the baseline analysis.^22^ Finally, we break down blood tests outcome to blood biochemistry and blood hematology [panels (e) and (f)]. The estimates again show that our findings are not driven by blood biochemistry tests, indicating that our results are associated with acute conditions.

### 3.6 Consequences of increased diagnostic tests utilization

Does the increased use of diagnostic tests in visits around decadal birthdays have consequences for subsequent treatment? The answer to this question may shed some light on the normative interpretation of the results. If the increase in the use of basic diagnostics in visits with unfamiliar patients is associated with changes in subsequent treatment and ultimately with improved patient outcomes, these additional tests are beneficial. On the other hand, if these tests are not associated with any change in the course of treatment and patient outcomes, they probably represent a waste of resources. We assess this question by examining whether visiting an unfamiliar physician just after a decadal birthday affected the likelihood of the following four outcomes: a subsequent visit to the clinic, a prescription for antibiotics, a referral to a specialist, and a referral to the ED (all within 60 days of the visit).^23^ This set of outcomes covers the relevant potential physician's responses to informative diagnostic tests results. If the increase in diagnostic tests affected treatment, it should show up in these subsequent treatment outcomes.

**FIGURE 6:**
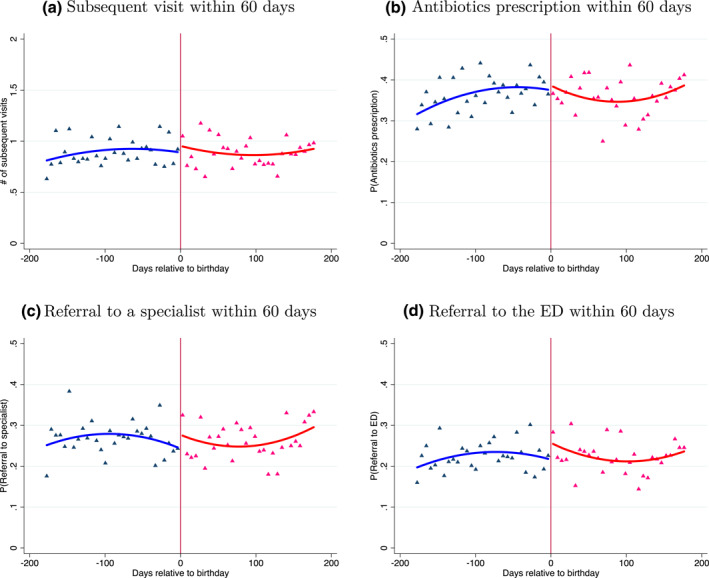
Subsequent outcomes around decadal birthdays, unfamiliar patients. Panels (a), (b), (c) and (d) of the figure plot the number of subsequent visits within 60 days, and the likelihood of receiving a prescription for antibiotics, a referral to a specialist or a referral to the ED by days elapsed relative to a patient's nearest decadal birthday, 180 days before and 180 days after the decadal birthday, in 6 days bins. The vertical solid line represents the decadal birthday threshold

We report the results in Figure 6 and Table 7. The impression that the four panels of Figure 6 create is that all four outcomes trend quite smoothly around the decadal age threshold. The estimates in Table 7 support this impression. None of the four outcomes significantly change around the decadal birthday threshold. These results suggest that the increase in the utilization of diagnostic tests around decadal birthdays does not induce changes in the course of treatment of affected patients.

**TABLE 7:** Outcomes of decadal birthdays, visits with unfamiliar patients

	Subsequent visit	Antibiotics	Referral to a specialist	Referral to the ED
	(1)	(2)	(3)	(4)
RDD estimate	−0.018	−0.023	0.012	0.019
	(0.075)	(0.029)	(0.024)	(0.020)
Bandwidth	35	28	34	42
Effective observations	1386	1103	1350	1683
Observations	7098	7098	7098	7098

*Note*: This table provides the RDD estimates for the analysis of subsequent outcomes of decadal birthdays. One or two asterisks indicate significance at 5% or 1%, respectively.

### 3.7 Non decadal birthdays

If our results arise from a left digit bias, visits with unfamiliar patients around birthdays that do not end with zero (“non decadal birthdays”) should show smaller effects. If, on the other hand, the results are an artifact of some other “birthday effect”, we may see a similar pattern around the birthdays that do not end with zero. We examine this by analyzing the utilization of basic diagnostic tests in all visits around non decadal birthdays using the same RDD approach we have used so far.

**FIGURE 7:**
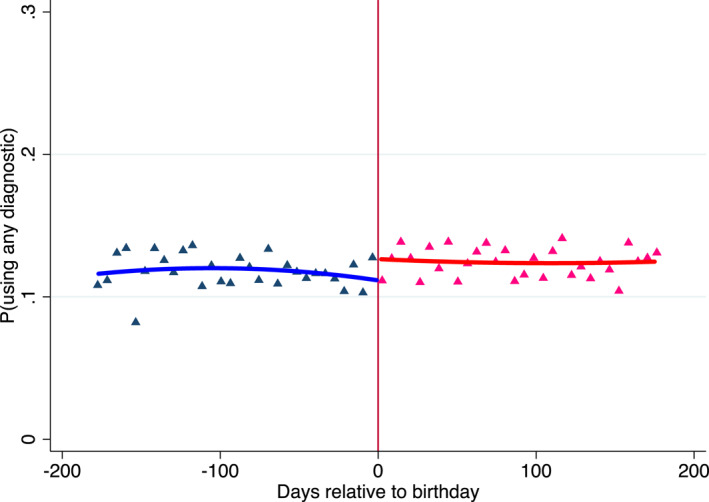
Utilization of basic diagnostic tests around non decadal birthdays, unfamiliar patients. The figure plots the likelihood of using any basic diagnostic test, by days elapsed relative to a patient's nearest non decadal birthday, 180 days before and 180 days after the decadal birthday, in 6 days bins. The vertical solid line represents the decadal birthday threshold

We show these results graphically in Figure 7. The utilization of basic diagnostic tests trends smoothly around non decadal birthdays. Consistent with this impression, the corresponding estimates, reported in Table 8, show no change in the utilization of basic diagnostic tests around non decadal birthdays. In columns (1)–(9) of Table 9, we perform the analysis separately for each of the non decadal birthdays ending in digits 1–9, respectively. We do not find statistically significant results for any of these birthdays.^24^

**TABLE 8:** The effect of non decadal birthdays, visits with unfamiliar patients

	(1)	(2)	(3)
RDD estimate	0.008	0.009	0.009
	(0.007)	(0.007)	(0.007)
Time FEs	No	Yes	Yes
Physician FEs	No	Yes	Yes
Patient characteristics	No	No	Yes
Bandwidth	31	31	31
Effective observations	9049	9049	9049
Observations	52,841	52,841	52,841

*Note*: This table provides the RDD estimates of the likelihood to use basic diagnostic tests as per Equation (3), for non decadal birthdays. Time fixed effects include year and month‐of‐year fixed effects. The patient characteristics which are included are: cubic polynomial of age, gender, and the following chronic conditions: hypertension, smoking, asthma, hyperlipidemia, obesity and diabetes. One or two asterisks indicate significance at 5% or 1%, respectively.

**TABLE 9:** The effect of non decadal birthdays, by the last digit

	One	Two	Three	Four	Five	Six	Seven	Eight	Nine
Age ending with	(1)	(2)	(3)	(4)	(5)	(6)	(7)	(8)	(9)
RDD estimate	0.025	−0.018	0.004	0.013	−0.012	0.004	−0.020	0.027	0.044
	(0.019)	(0.022)	(0.021)	(0.022)	(0.016)	(0.020)	(0.027)	(0.022)	(0.029)
Bandwidth	26	23	29	26	44	34	21	32	23
Effective observations	940	827	1011	878	1455	1079	622	893	634
Observations	6827	6579	6395	6072	6060	5752	5275	5045	4836

*Note*: Columns (1)‐(9) of this Table 9 provide the RDD estimates of the likelihood to use basic diagnostic tests as per Equation (3), separately nine times of each of the non‐decadal birthdays ending in digits 1‐9, respectively. One or two asterisks indicate significance at 5% or 1%, respectively.

This analysis shows that visits with unfamiliar patients around non decadal birthdays do not result in a significant increase in the utilization of basic diagnostic tests, indicating that the results we find for visits around decadal birthdays are unique. These results support the view that the increase in the utilization of basic diagnostic tests around decadal birthdays indeed arises from physicians' left digit bias with respect to the patient's age.

## 4 CONCLUSION

Do PCPs use simplifying heuristics in their clinical decision‐making? Concretely, this paper examines whether PCPs exercise the so‐called left digit bias with respect to patients' age. We rely on comprehensive administrative visit level data from a large Israeli HMO to examine the utilization of basic diagnostic tests in visits that take place around a decadal birthday within a regression discontinuity framework. In essence, we examine if there is evidence that visits that occur just after a decadal birthday are associated with more utilization of basic diagnostic tests.

This study provides new evidence on the nature of heuristic thinking by PCPs. It shows that in settings with longitudinal physician‐patient relationships and clear patient information, there is no evidence that PCPs exhibit left digit bias. However, when PCPs meet sporadically unfamiliar patients seeking immediate care, they exercise left digit bias with respect to the patient's age. Hence, the evidence suggests that heuristic thinking tends to emerge in relatively confusing and opaque circumstances with scarce background information. This result sheds light on the impact of decision‐making settings on expert choices in medicine and beyond.

A priori, it is not clear if the increase in diagnostic tests is efficient. The decadal age threshold may operate as a naturally occurring reminder about the patient's age inducing the physician to order the “age‐appropriate” amount of diagnostic tests. While we cannot determine conclusively whether the additional diagnostic tests indicate “waste”, we find no evidence of impact on subsequent treatment. If one is willing to take our results at face value, then from a health care policy perspective, our findings can be viewed as evidence in favor of developing physician‐patient relationships and care continuity in the primary care setting. The longitudinal physician‐patient relationship appears to alleviate some cognitive burden and reduce the tendency to use heuristics.

## CONFLICT OF INTEREST

The author has no conflict of interest to declare.

## Supporting information

Supporting Information S1

## Data Availability

Data available on request due to privacy/ethical restrictions. The data used in this research are proprietary. This means that should the data be requested by a reader, we will be able to make it available provided the owners of the data agree to this.

## ENDNOTES

^1^
See for example, Chan Jr et al. (2019), Currie and MacLeod (2020), Chandra and Staiger (2020), Abaluck et al. (2016) and Mullainathan and Obermeyer (2019).

^2^
Gabaix (2017) provides a recent review of this topic.

^3^
See also https://stats.oecd.org/. More than 40% of physician workforce in a number of high‐income countries is comprised by primary care physicians (Papanicolas et al., 2018).

^4^
The data include the physician identity as well as the patient's regular physician identity allowing us to distinguish between visits with a patient's regular physician and visits with another physician.

^5^
These results are derived using a zero‐order local polynomial regression with MSE optimal bandwidth, but we find that they are not sensitive to the bandwidth, degree of the polynomial function, and the kernel function we use. We also test the continuity of the density of visits around the decadal birthday threshold and find no evidence of such a pattern nor for “selection on observables”.

^6^
see Chandra et al. (2011) and Saposnik et al. (2016) for reviews of existing literature on behavioral aspects of physician decision making.

^7^
This study also contributes to the existing literature about the so‐called left digit bias in other contexts. For example, Lacetera et al. (2012), Repetto and Solís (2017), Ater and Gerlitz (2017) and Shlain (2018) (see also the literature review on this issue in that paper).

^8^
The header includes some basic patient information such as date of birth, gender, address, and phone number.

^9^
To reiterate, in our context, this is *not* a decimal: 11 stands for 11 months and not 0.11 of a year.

^10^
Every Israeli resident may freely choose an HMO. The Israeli health care system is largely publicly funded, with much of the HMOs' budget being derived from health taxes.

^11^
The number of physicians seeing unfamiliar patients is larger because, by definition, a temporary physician would only appear in that group, while permanent physicians should appear in both groups. However, these 19 physicians account for only 313 observations. Excluding them from the sample does not change the results.

^12^
Visits with unfamiliar patients include encounters with physicians who substitute the regular physician for an extended time, for example, if the regular physician is on maternity leave. Thus, in some cases, patients do have prior contact with that physician.

^13^
Appendix Figure A1 in the Supporting Information S1 shows this by visit type.

^14^
The analysis of the joint sample—unfamiliar and familiar patients appears in Appendix Figure A2 and Appendix Tables A1 and A2 in the Supporting Information S1.

^15^
In Appendix Figure A4 in the Supporting Information S1, we present a full “scatter plot” of the data, the likelihood of using basic diagnostic tests in each day relative to the decadal birthday. Figure A3 in the Supporting Information S1 provides another version of the figure with the “optimal” quantile‐spaced bins using Integrated Mean Squared Error (IMSE) method (Calonico et al., 2015).

^16^
Table A3 in the Supporting Information S1 shows the results for using a first‐order polynomial, which are slightly larger.

^17^
The results are not sensitive to the choice of the kernel function we apply. Epanechnikov and uniform kernels provide very similar results.

^18^
This is often the case in RDD designs Cattaneo et al. (2017).

^19^
We set the bandwidth in all the regressions according to the optimal bandwidth in the unfamiliar group—39 days around a decadal birthday.

^20^
Time fixed effects include year and month‐of‐year fixed effects. The patient characteristics included are cubic polynomial of age, gender, and the following chronic conditions: hypertension, smoking, asthma, hyperlipidemia, obesity, and diabetes—the same patient‐level variables we use in the selection‐on‐observables analysis.

^21^
See for example, Barreca et al. (2016).

^22^
Appendix Figures A7 and A8 in the Supporting Information S1 display the RDD figures that correspond to the analysis of each of the outcome variables we report in Table 6.

^23^
Ideally, one would want to include long term outcomes, but those are not available to us.

^24^
Birthdays that end with nine exhibit a relatively large point estimate. However, the result is insignificant and it tends to zero as the bandwidth increases.
