# Supplementary material for: Heuristic thinking in the workplace: Evidence from primary care
Source: Health Econ. 2022 May 23;31(8):1713–29. doi: 10.1002/hec.4534 (PMC9540444; doi:10.1002/hec.4534)
Supplement: Supplementary file 1 — Supporting Information S1 [file HEC-31-1713-s001.pdf]

A Appendix A

Figure A.1: Share of diagnostic tests by age, by visit-type

(a) Unfamiliar patients

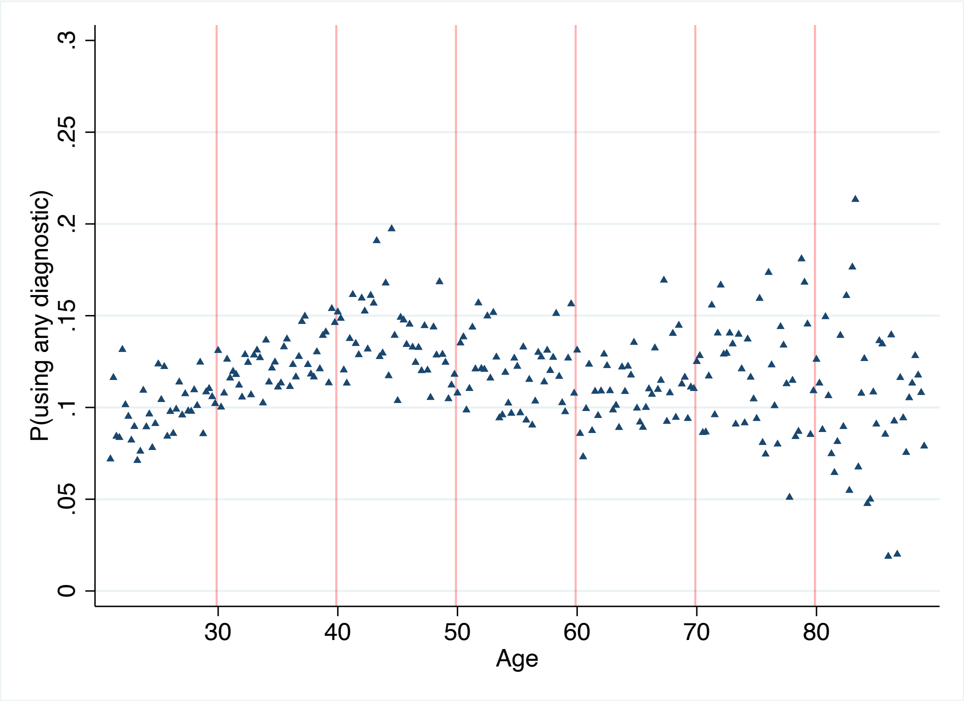

(b) Familiar patients

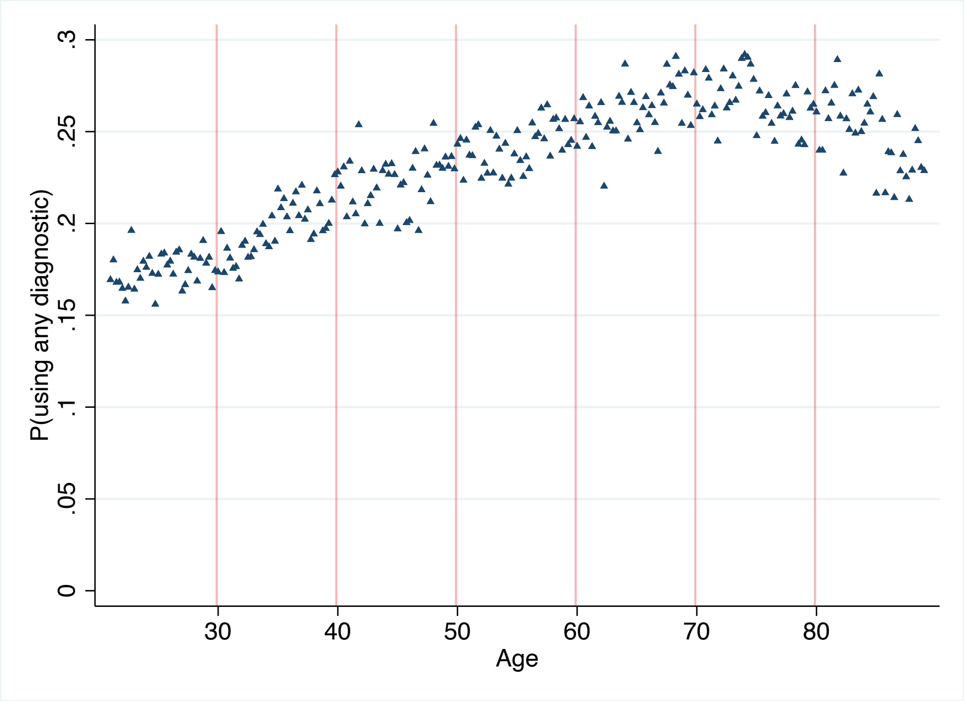

Note: Panels (a)-(b) plot the share of visits with any basic diagnostic tests by age in quarters, for visits with unfamiliar and familiar patients, respectively.

Figure A.2: Utilization of basic diagnostic tests around non decadal birthdays, joint sample of unfamiliar and familiar patients

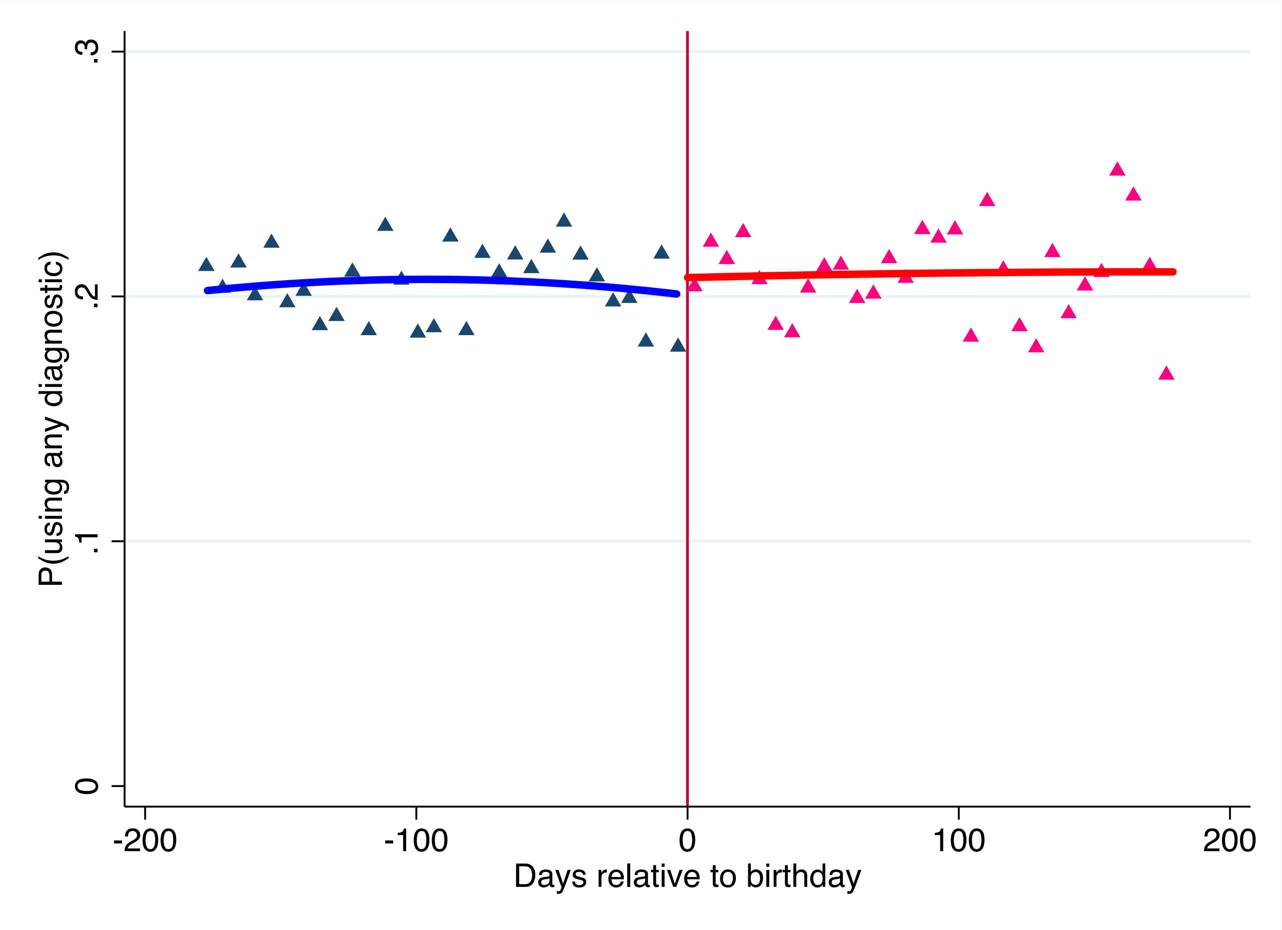

Note: The figure plots the likelihood of using any basic diagnostic test, by days elapsed relative to a patient's nearest non decadal birthday, 180 days before and 180 days after the decadal birthday, in six day bins. The vertical solid line represents the decadal birthday threshold.

Figure A.3: Utilization of basic diagnostic tests around decadal birthdays, optimal bin width

(a) Unfamilar patients

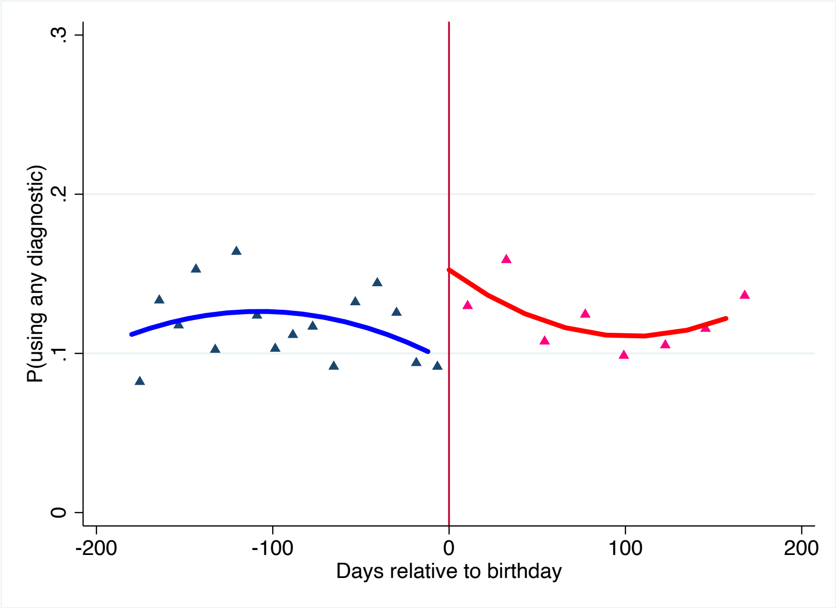

(b) Familar patients

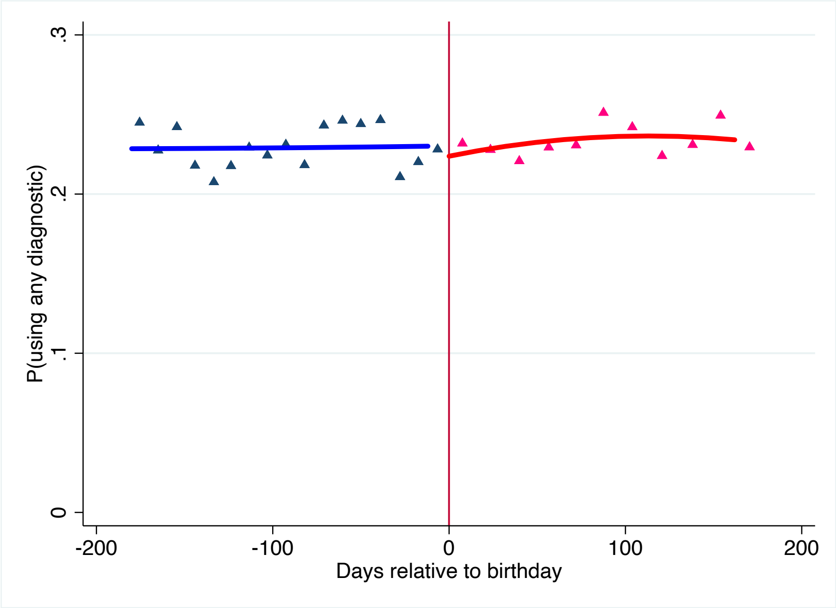

Note: Panels (a) and (b) of the figure plot the likelihood of using any basic diagnostic test, by days elapsed relative to a patient's nearest non decadal birthday, 180 days before and 180 days after the decadal birthday, with optimal quantile-spaced bins using Integrated Mean Squared Error (IMSE) method (Calonico et al. (2015)). The vertical solid line represents the decadal birthday threshold.

Figure A.4: Utilization of basic diagnostic tests around decadal birthdays, scatter plot

(a) Unfamilar patients

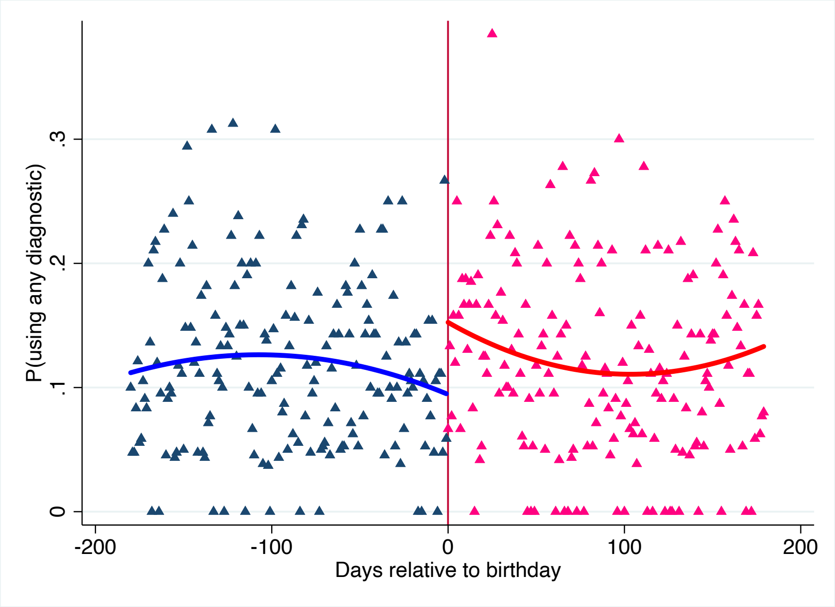

(b) Familar patients

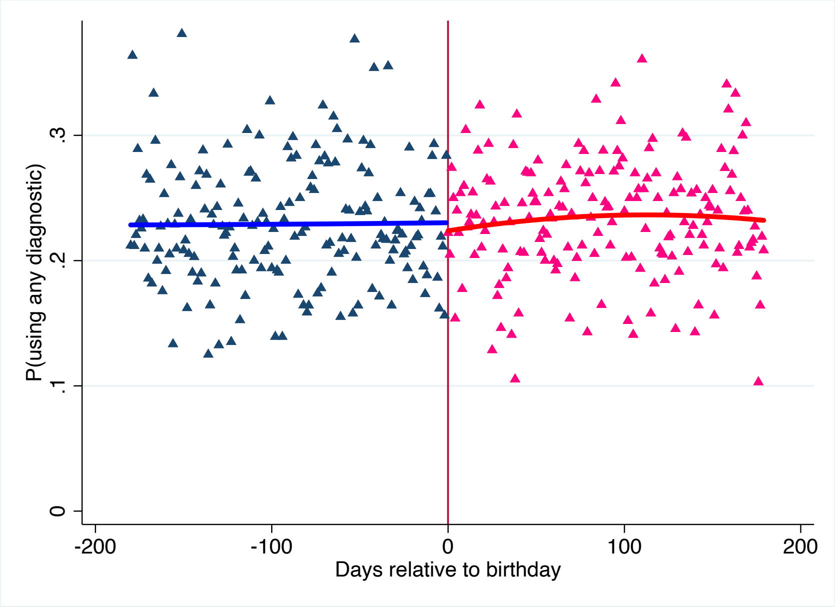

Note: Panels (a) and (b) of the figure plot the likelihood of using any basic diagnostic test, by days elapsed relative to a patient's nearest non decadal birthday, 180 days before and 180 days after the decadal birthday, in single day bins. The vertical solid line represents the decadal birthday threshold.

Figure A.5: Selection on observables, visits with unfamiliar patients

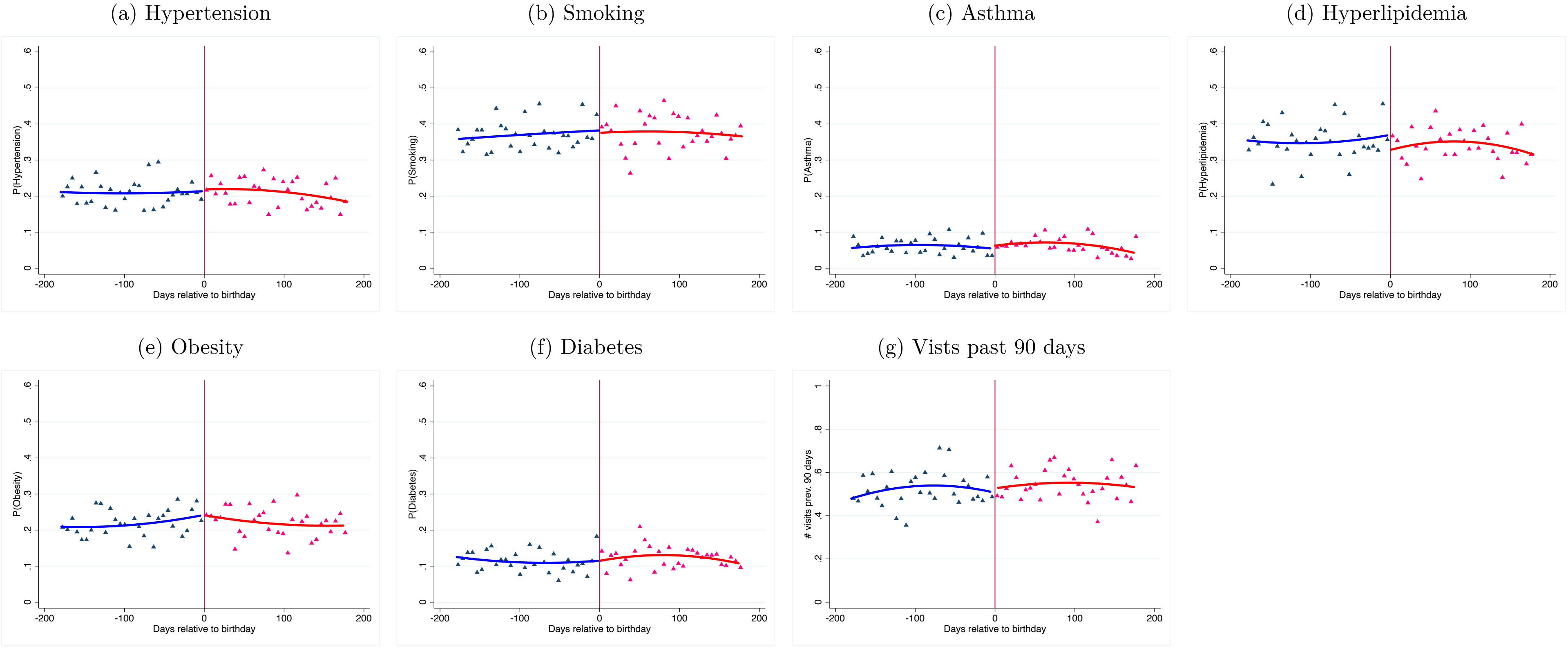

Note: Panels (a)-(g) of the figure show how observable visit characteristics trend around the decadal birhtday threshold. All panels depict variable averages by days elapsed relative to a patient's nearest decadal birthday, 180 days before and 180 days after the decadal birthday, in six days bins, 30 bins on each side of the threshold. The vertical solid line in all panels represents the decadal birthday threshold.

Figure A.6: Selection on observables, visits with familiar patients

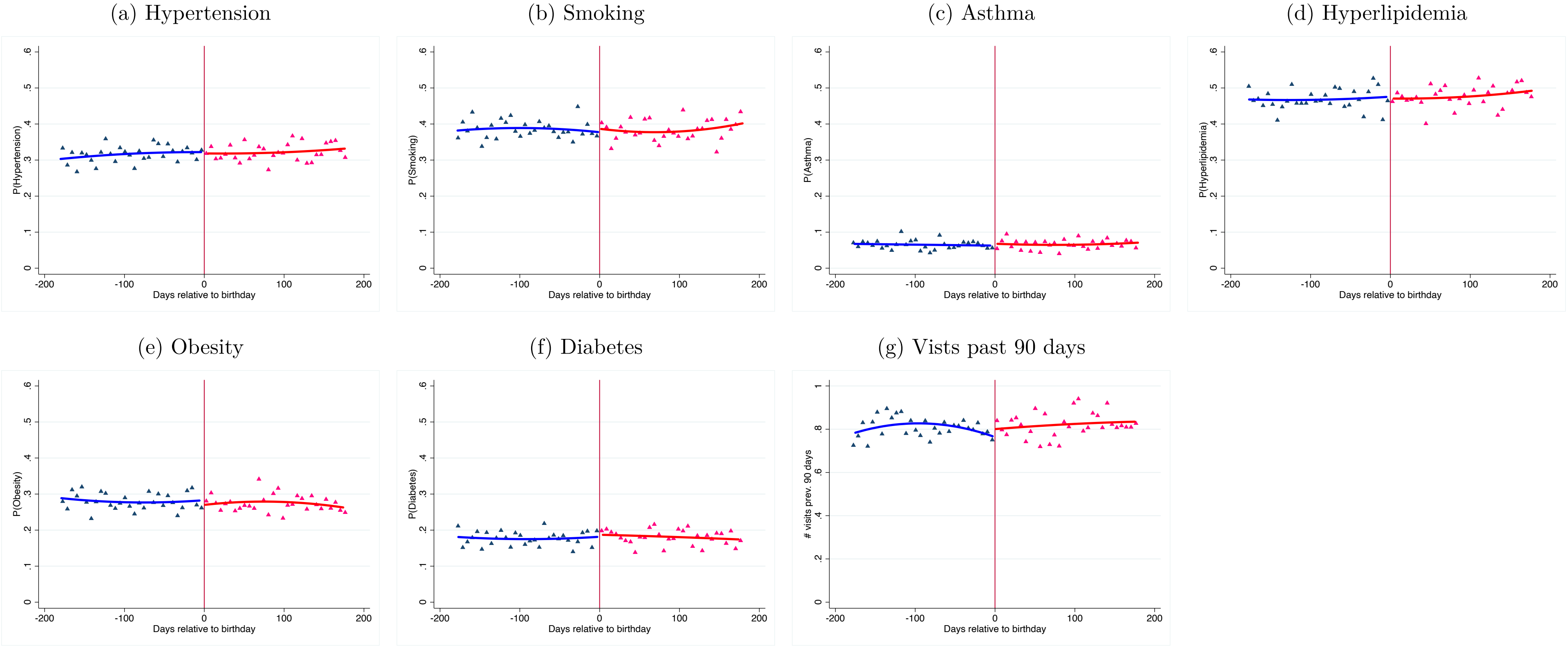

Note: Panels (a)-(g) of the figure show how observable visit characteristics trend around the decadal birthday threshold. All panels depict variable averages by days elapsed relative to a patient's nearest decadal birthday, 180 days before and 180 days after the decadal birthday, in six days bins, 30 bins on each side of the threshold. The vertical solid line in all panels represents the decadal birthday threshold.

Figure A.7: Utilization of diagnostic tools around non decadal birthdays, visits with unfamiliar patient

(a) Diagnostics no bio-chem

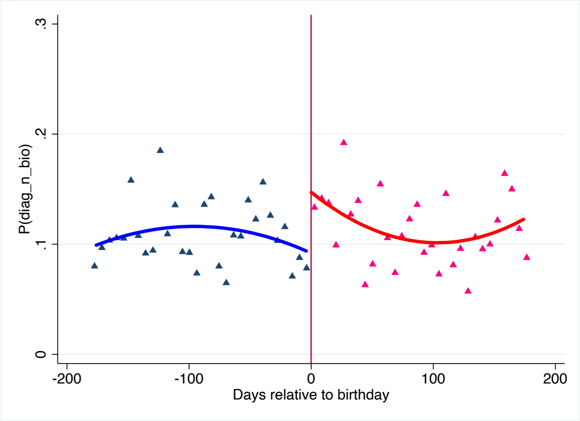

(b) X-ray

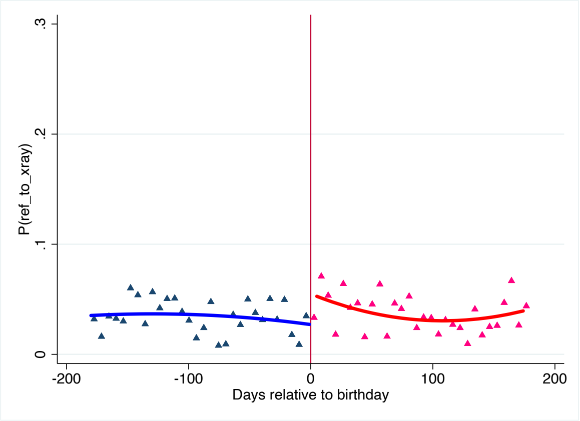

(c) Urine test

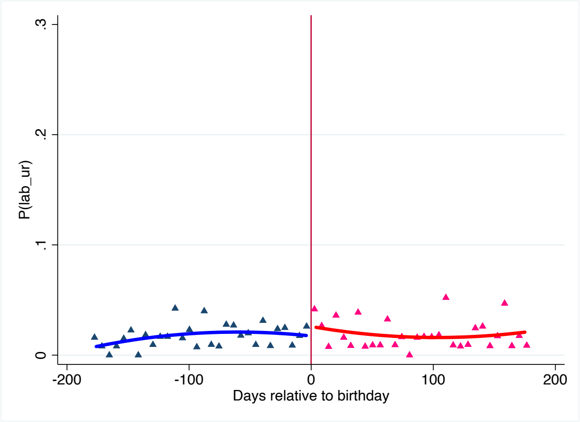

(d) Blood test

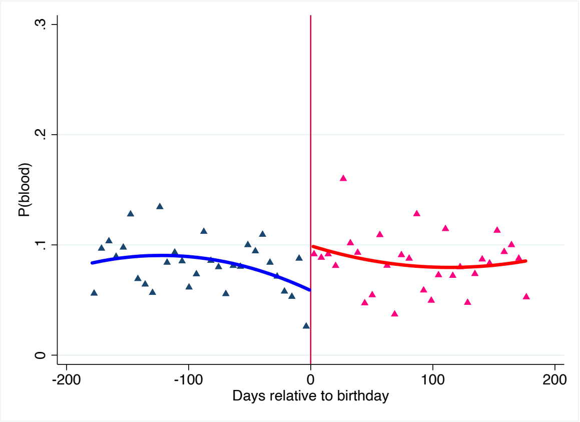

(e) Blood no bio-chem

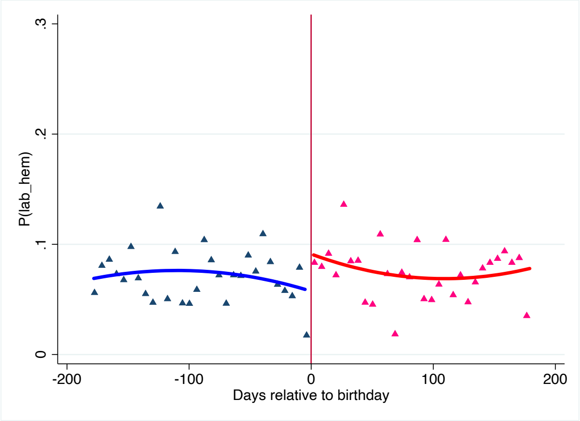

(f) Blood bio-chem

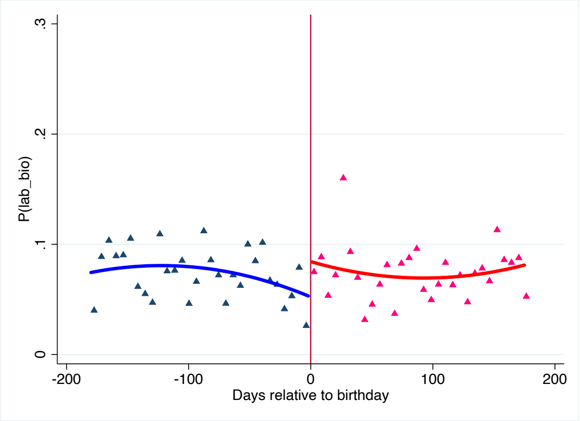

Note: Panel (a) of the figure shows the result of the main outcome excluding bio-chem blood tests. Panels (b)-(d) plot the likelihood of using the components of basic diagnostic tools: X-ray, blood test, and urine test. Panels (e)-(f) show the breakdown of blood tests into two types: bio-chem and no bio-chem (hematology). All panels show results for days elapsed relative to a patient's nearest non decadal birthday, 180 days before and 180 days after the decadal birthday, in 6 day bins. The vertical solid lines represent the decadal birthday threshold.

Figure A.8: Utilization of diagnostic tools around non decadal birthdays, visits with familiar patient

(a) Diagnostics no bio-chem

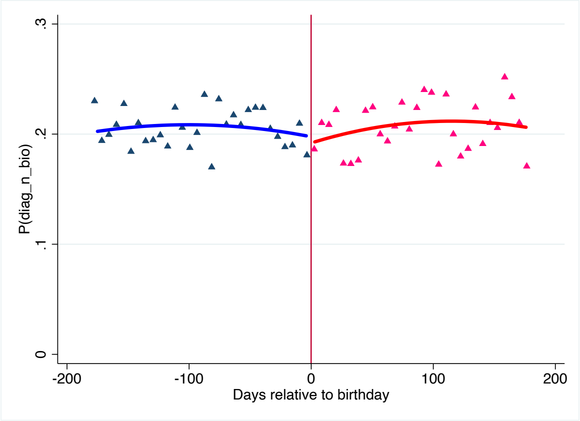

(b) X-ray

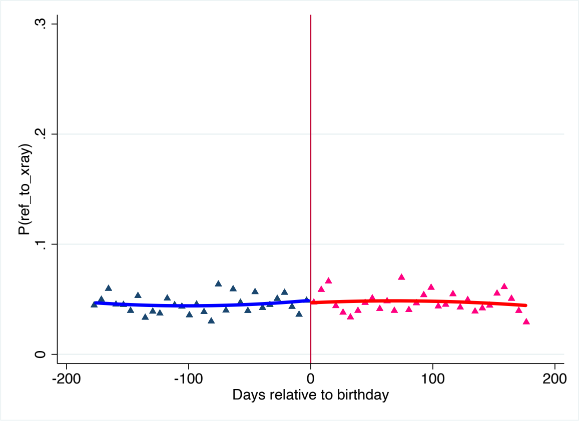

(c) Urine test

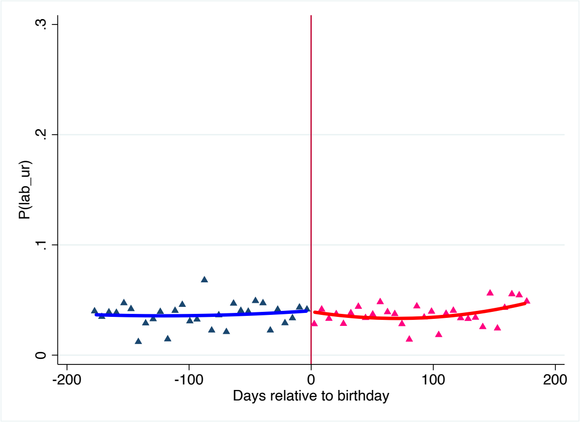

(d) Blood test

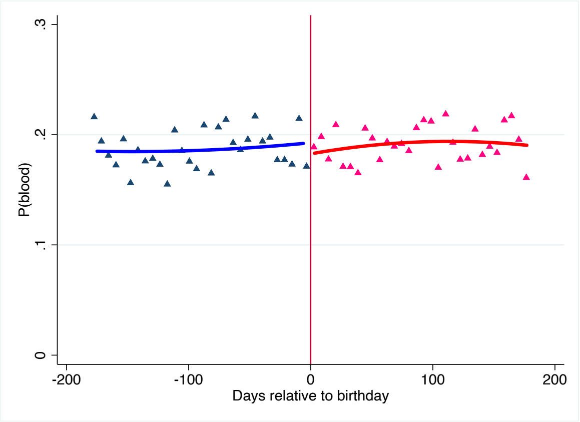

(e) Blood no bio-chem

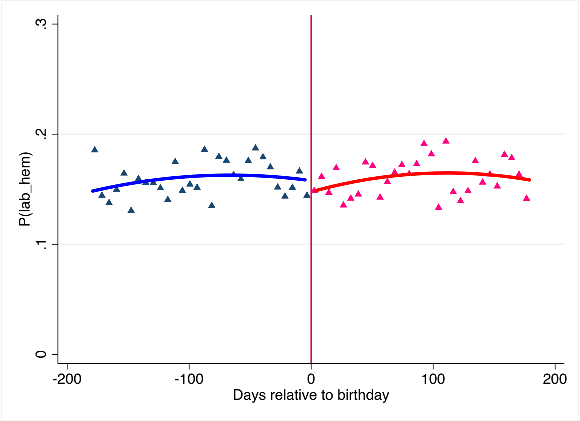

(f) Blood bio-chem

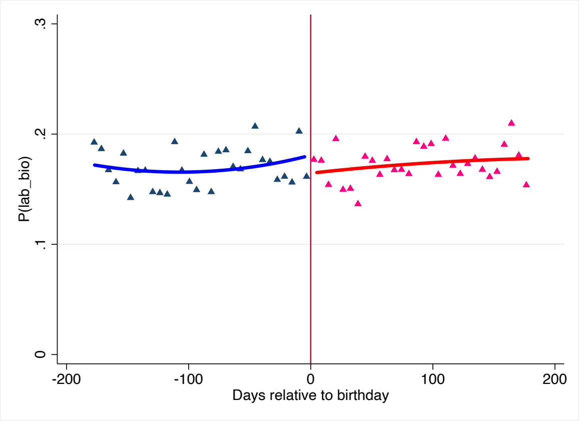

Note: Panel (a) of the figure shows the result of the main outcome excluding bio-chem blood tests. Panels (b)-(d) plot the likelihood of using the components of basic diagnostic tools: X-ray, blood test, and urine test. Panels (e)-(f) show the breakdown of blood tests into two types: bio-chem and no bio-chem (hematology). All panels show results for days elapsed relative to a patient's nearest non decadal birthday, 180 days before and 180 days after the decadal birthday, in 6 day bins. The vertical solid lines represent the decadal birthday threshold.

Table A.1: Summary statistics joint sample of unfamiliar and familiar patients

	Mean (1)	SD (2)
Mean age	51.30	16.66
Share women	0.58	0.49
Share born in Israel	0.69	0.46
Share hypertension	0.30	0.46
Share smokers	0.38	0.49
Share hyperlipidemia	0.45	0.50
Share asthma	0.06	0.25
Share overweight	0.26	0.44
Share diabetes	0.17	0.37
Share referral to X-ray	0.04	0.21
Share referral to blood test	0.14	0.35
Share referral to urine test	0.03	0.18
Share referral to basic diagnostic test	0.21	0.41
Share upper respiratory infection acute	0.03	0.16
Share upper respiratory tract infection	0.02	0.14
Share viral infection unspecified	0.02	0.13
Share visit in prior 360 days	0.66	0.47
Share visit any physician in prior 90 days	0.44	0.50
Number of patients	15,280	
Number of physicians	97	
Observations	32,793	

Notes: The table includes office visits in the clinics used in this study in the period 2011-2014.

Table A.2: The effect of decadal birthdays on utilization of basic diagnostic tests, joint sample

	(1)	(2)	(3)
RDD Estimate	0.017	0.015	0.015
	(0.013)	(0.013)	(0.013)
Time FEs	No	Yes	Yes
Physician FEs	No	Yes	Yes
Patient characteristics	No	No	Yes
Bandwidth	22	22	22
Effective observations	3,948	3,948	3,948
Observations	32,793	32,793	32,793

Note: This table provides the RDD estimates of the likelihood to use basic diagnostic tests as per Equation 3. Time fixed effects include year and month-of-year fixed effects. The patient characteristics which are included are: cubic polynomial of age, gender, and the following chronic conditions: hypertension, smoking, asthma, hyperlipidemia, obesity and diabetes. One or two asterisks indicate significance at 5% or 1%, respectively.

Table A.3: The effect of decadal birthdays, first order polynomial

	1st order polynomial		
	Familiar (1)	Unfamiliar (2)	Diff (3)
RDD Estimate	0.056*	0.020	0.036
	(0.026)	(0.018)	(0.036)
Bandwidth	48	48	48
Effective observations	1,916	6,743	8,659
Observations	7,098	25,695	32,793

Note: This table provides the RDD estimates of the likelihood to use basic diagnostic tests as per Equation 3. Time fixed effects include year and month-of-year fixed effects. The regression uses 1st order polynomial local regression. The patient characteristics which are included are: cubic polynomial of age, gender, and the following chronic conditions: hypertension, smoking, asthma, hyperlipidemia, obesity and diabetes. One or two asterisks indicate significance at 5% or 1%, respectively.

B Appendix B

B.1 Heterogeneity analysis by patient age

Our analysis pools together the effect of decadal birthdays on physician behavior around different ages. However, the effect may vary by patient age, depending on the importance of the patient's age as a risk factor per se. It is not a priori obvious whether the perception of age, given other medical examination results and the patient's condition, plays a more important role in PCP decision-making in younger or older patients.

We examine this issue by splitting the sample into two age groups: 30-50 and 60-80. We perform an RDD analysis, similar to the analysis above, for each age group separately and report the results in Figure B.1 and Table B.1. As the figure shows, the increase in the likelihood of using basic diagnostics exists in both groups but it appears to be sharper in the age 30-50 group. This impression is consistent with the estimates in Table B.1. The result for the age 30-50 group – in column (1) – is (a significant) 7 pp increase. The result in the age 60-80 age group are 2.2 percentage points and they are statistically insignificant. We note that it would be ideal to further break down the data by each decade but this is hindered by statistical power limitation. Nonetheless, we plot the data for each decadal birthday in Appendix Figure B.2. The impression from this figure, which is naturally quite noisy, is that the utilization of basic diagnostic testing shows an apparent jump above the decadal birthday thresholds except around ages 50 and 80.

Figure B.1: Utilization of basic diagnostic tests around decadal birthdays, by age, unfamiliar patients

(a) Decadal birthdays 30-50

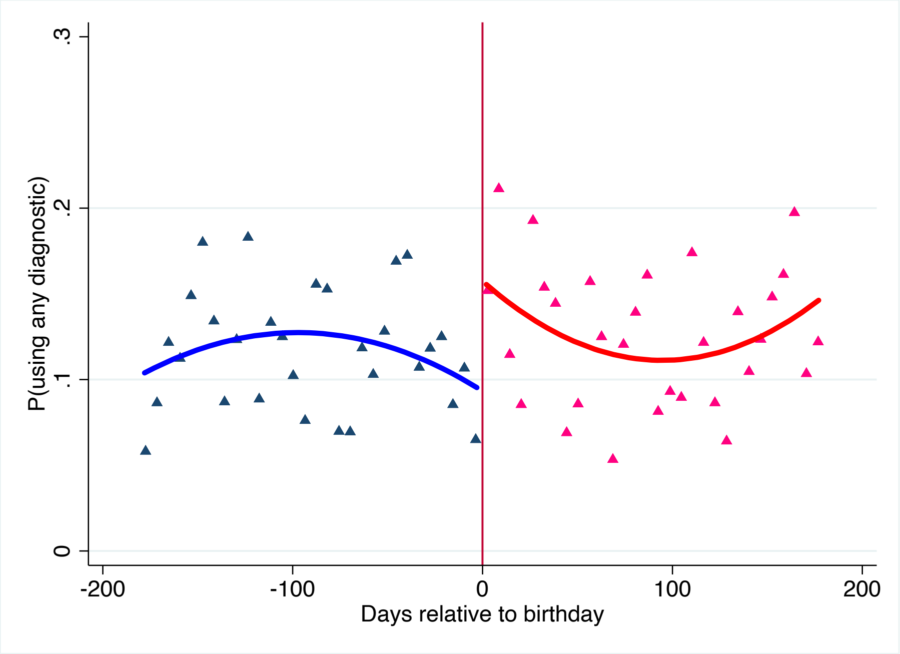

(b) Decadal birthdays 60-80

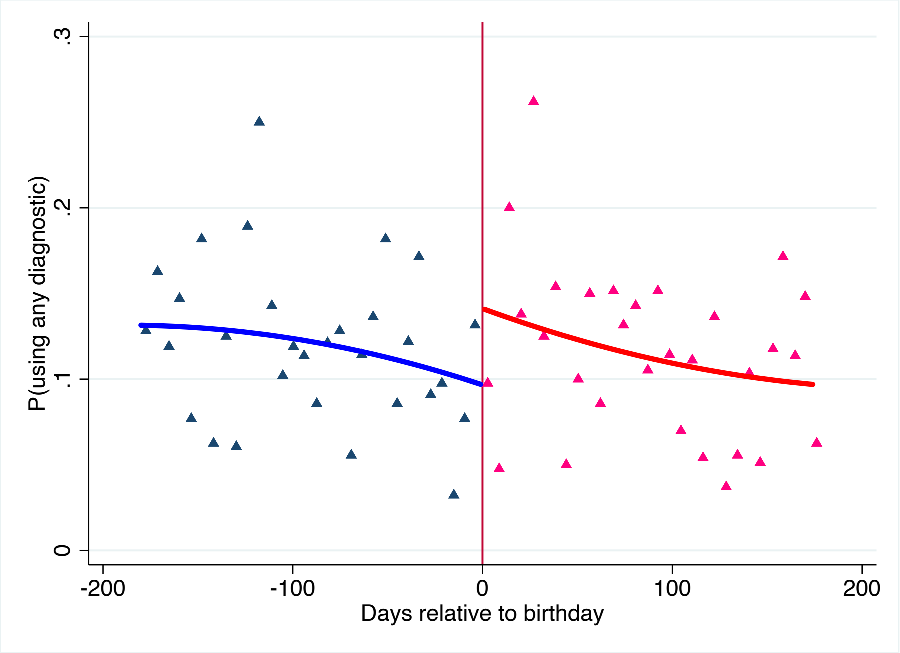

Note: Panels (a)-(b) of the figure plot the likelihood of using any basic diagnostic test, by days elapsed relative to a patient's nearest decadal birthday, 180 days before and 180 days after the decadal birthday, in six days bins, by patients' age. The vertical solid line represents the decadal birthday threshold.

Figure B.2: Utilization of basic diagnostic tests around decadal birthdays by decadal age

(a) Age 30

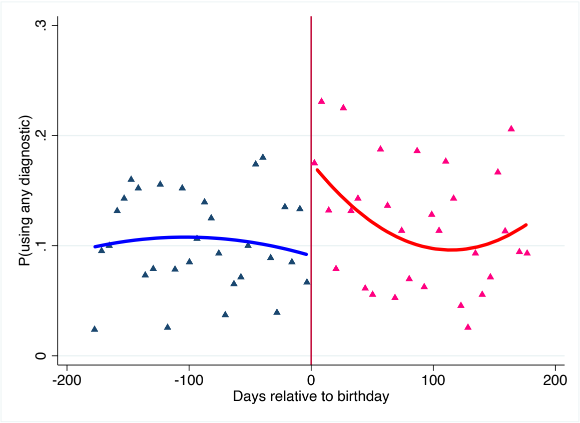

(b) Age 40

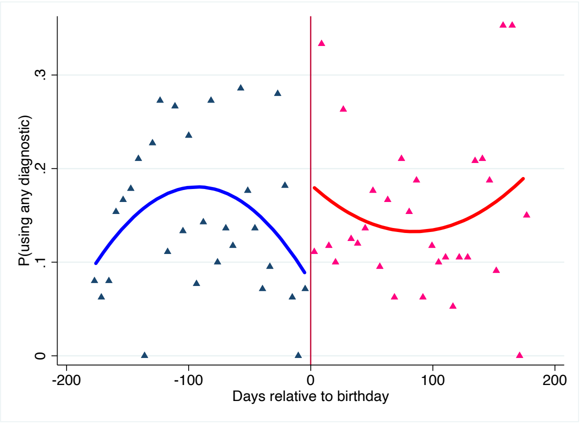

(c) Age 50

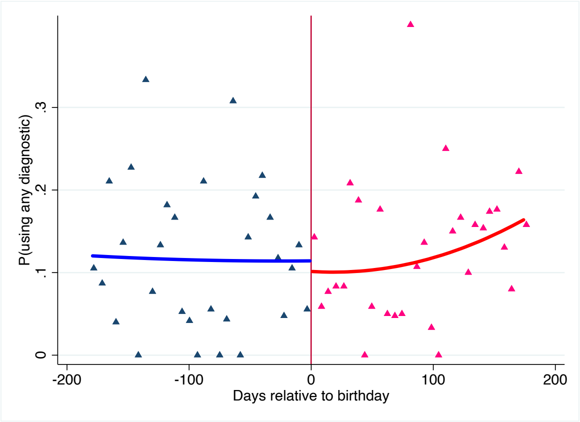

(d) Age 60

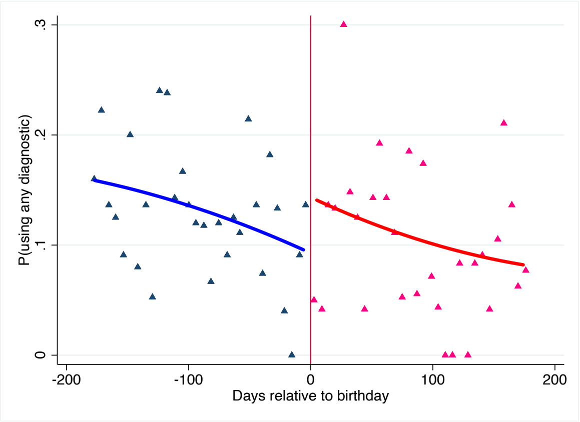

(e) Age 70

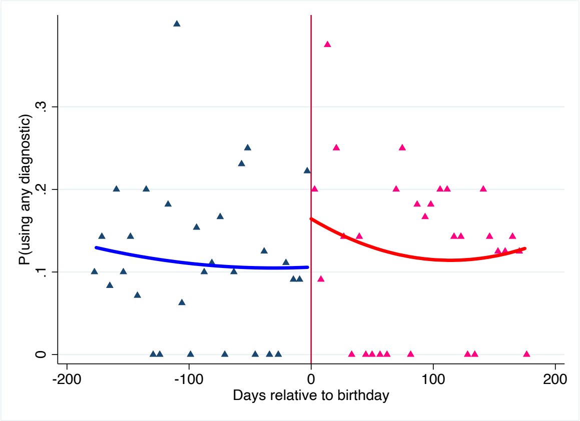

(f) Age 80

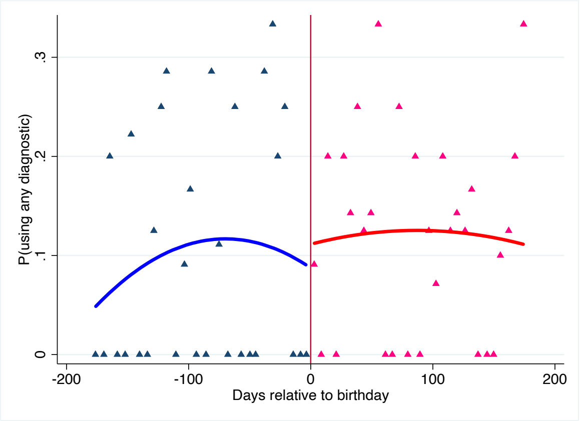

Note: Panels (a)-(f) of the figure plot the likelihood of using any basic diagnostic test, by days elapsed relative to a patient's nearest non decadal birthday, 180 days before and 180 days after the decadal birthday, in 6 day bins. The vertical solid lines represent the decadal birthday threshold.

Table B.1: The effect of decadal birthdays, by patient age

	B-days 30,40,50	B-days 60,70,80
	(1)	(2)
RDD Estimate	0.068*	0.021
	(0.027)	(0.032)
Bandwidth	22	31
Effective observations	586	383
Observations	4,881	2,217

Note: This table provides the RDD estimates of the likelihood to use basic diagnostic tests as per Equation 3, by patient age. One or two asterisks indicate significance at 5% or 1%, respectively.
